# A checklist of spiders of Nepal (Arachnida; Araneae)

**DOI:** 10.1016/j.heliyon.2022.e09927

**Published:** 2022-07-13

**Authors:** Sanskar Subedi, Ritu Joshi, Samir Karki, Shila Gurung

**Affiliations:** Institute of Forestry, Pokhara Campus, Nepal

**Keywords:** Endemic, Nepalese, Species, Spider

## Abstract

Spiders (order Araneae) have a worldwide distribution. As of June 17, 2022, the World spider catalog, Version 23.0, enlists 50,188 species of spiders from throughout the world. Except for the open sea and air, spiders live in every ecological environment. They prefer densely vegetated environments and are the world's seventh most diverse group of creatures in terms of species diversity. The checklist of spiders of south Asia mentions 222 spider species from Nepal, grouped into 23 families. However, the official database of the Nepalese government only lists 175 species of Nepalese spiders. This checklist is a vital update to the diversity of the spider fauna of Nepal. The goal is to compile a thorough list of all the spiders found in Nepal. We have enlisted 386 different spider species from Nepal, belonging to 135 different genera and 34 different families, after reviewing previous scientific publications, computer databases of the Nepalese government, and the World spider catalog, Version 23.0. It adds a total of 211 new spiders to Nepal's biodiversity database. Linyphiidae and Salticidae spiders dominate accounting for 27.46 percent and 17.36 percent of total species, respectively, on the checklist. Corrections to previous misidentifications are also included in this checklist, as well as taxonomy revisions. Synonymous species are sorted out to avoid recurrence. The trends in Nepalese spider discovery and dispersion have also been studied.

## 1 Introduction

Spiders are found all over the planet and have mastered all natural situations, excluding the open sea and the air [1]. The World spider catalog, Version 23.0, has 50,188 species of spiders from all across the world as of June 17,2022 [2]. The amount of extant species of spiders has been estimated to be over 170,000 [3] although it could be much higher. Spiders are without a doubt the largest taxonomic group that is totally made up of predators. Part of their success can be attributed to their skill to colonize almost all terrestrial habitats, from marine intertidal zones [4]to high altitude areas, as evidenced by the champion *Euophrys omnisuperstes* [5] which can be found at 6700 m altitude in the Himalayas [6]. Spider study is growing, thanks to new taxonomic discoveries, neuronal properties of spider venom, and the versatility of spider silk.

Brignoli [7, 8, 9, 10], Wunderlich [11, 12, 13], Ono [14, 15, 16, 17, 18, 19], Zabka [20, 21, 22, 23], Bohdanowicz [24, 25], Thapa [26], and Buchar [27, 28, 29] were among the significant contributors to the study of Nepalese spiders in the mid-twentieth century [30]. Several studies have been based on Prof. J. Martens' collections from the Himalayan expeditions. Buchar introduced 7 new Lycosid spiders from Nepal in 1976, 1978, and 1984. Bohdanowicz (1979, 1987) described new *Synagelides* spiders from Nepal in his studies. Ono (1979, 1980, and 1985) used numerous thomisid spiders from Nepal in his research. Other researches by Ono (1983, 2006) and Jocqué (1992) describe endemic Zodariidae spiders from Nepal. Nishikawa's study from 1980 introduced 2 new *Agelena* spiders from Nepal's Khumbu region. Zabka discovered 11 distinct salticid spiders in Nepal during his research. Other 2 spider species belonging to the genus *Suffasia* were also discovered from eastern Nepal [31]. Two hersilid spiders from Nepal were introduced by Baehr & Baehr in 1993. There were 144 species of spiders in Nepal, according Thapa’s book; “Enumeration of Spiders of Nepal” [26]. Jastrzebski [32, 33, 34, 35, 36, 37, 38, 39] cites 15 different salticid spiders from Nepal in his various researches. A study on rice field spiders was also conducted in Nepal's Chitwan district [40]. Jäger [41, 42, 43, 44, 45] describes the family Sparassidae, which includes 33 species from Nepal. Wang discovered 7 new *Himalcoelotes* species in his research [46]. There are 37 new spiders of the genus *Draconarius* in the revised list of ceolotine spiders from Nepal [47]. Different Studies by Tanasevitch [48, 49, 50, 51, 52, 53, 54, 55, 56] Tanasevitch and Saaristo [57], and Wunderlich [11, 12, 13] enlist many linyphid spiders from Nepal. The “Nepal biodiversity resource book” featured a checklist of spiders of Nepal (Annex 2.1) with 175 species of spiders belonging to 22 distinct families, based on data from earlier publications [26, 40, 58]. Similarly, the checklist of south Asian spiders identified 222 spider species belonging to 79 genera. 176 of those species were only found in Nepal [30]. Wang and Zhu discovered 3 new species of the genus *Himalmartensus* in Nepal [59]. 38 different Nepalese spiders of the genus *Draconarius* are described in a study based on J. Marten's collection from Himalayan trips [47]. Four new *Camptoscaphiella* species were discovered in Nepal by Baehr and Ubick in 2010. Platnick et al. found 3 new species of *Brignolia* in Nepal [60]. Huber described 2 species of genus *Pholcus* from Nepal in 2011. Jager found a new species of genus *Ctenus* in 2012. Four species of the genus *Himalayana* from Nepal are included in Grismado’s study [61].

Nepal's official spider species count is 175 as published in “Nepal's Sixth National Report to The Convention on Biological Diversity” (2018), released by the Ministry of Forest and Environment, Government of Nepal [62]. Furthermore, the study by Xu et al. documented 5 new species of the genus *Leclercera* from Nepal [63]. Chang and Li added another *Leclercera* spider to the list [64]. For the first time in Nepal, Shrestha and Dorr announced the finding of the genus *Lactrodectus in* 2020 [65]. Nine new Asian salticid spider species were discovered during a field study in Nepal's Chitwan National Park [66]. A new genus, *Himalafurca*, is described in a recent study including two species from Nepal [52]. A different study reports 7 new *Pimoa* species from Nepal [67].

Furthermore research on Nepalese spiders has been ongoing since last inventory, and this update to the Nepalese spider checklist is critical. The purpose of this article is to compile a list of all the spider species discovered in Nepal, update their taxonomy and reveal the current state of spider research in Nepal.

## 2 Study area

**Figure 1:**
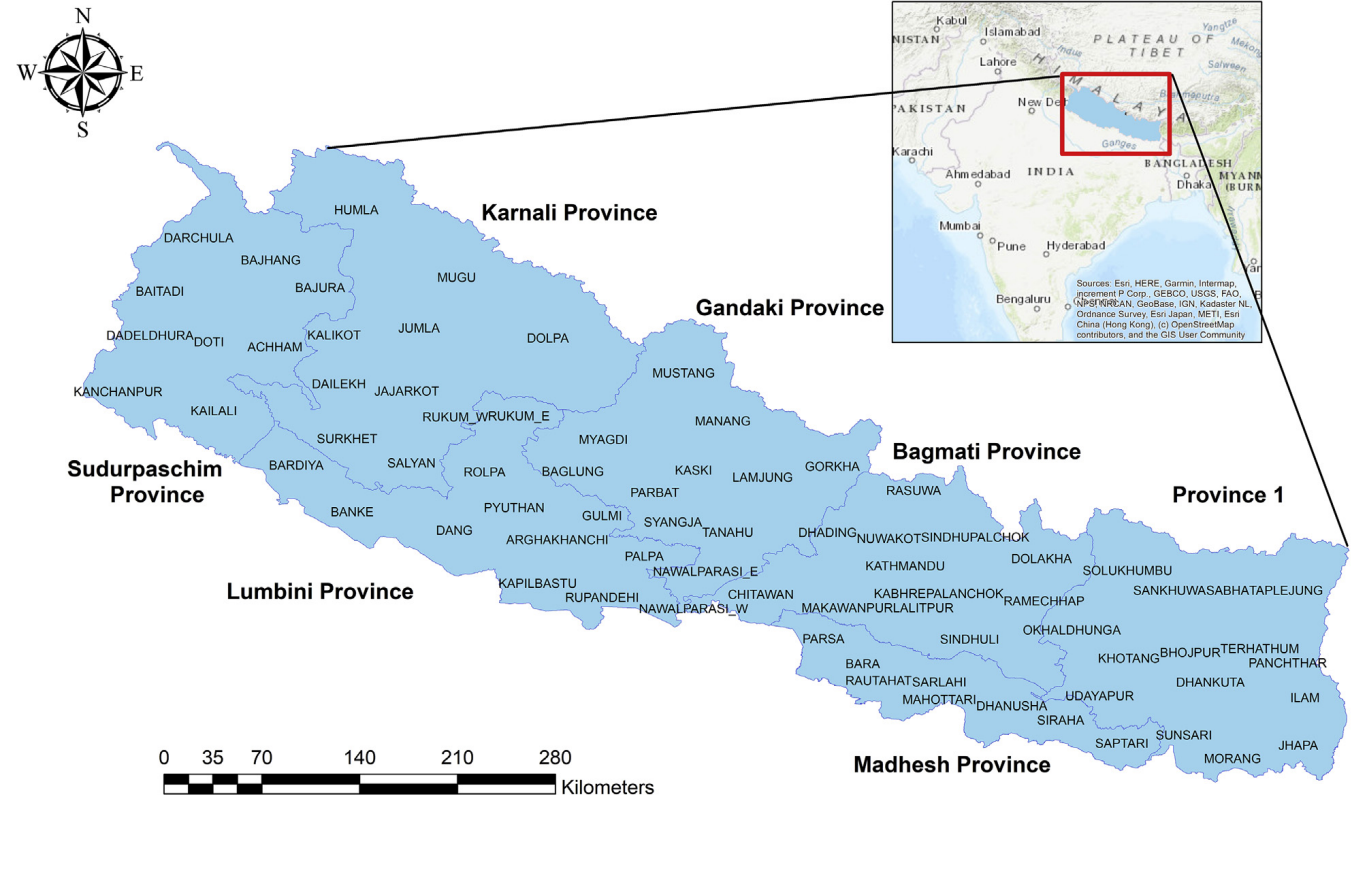
Map of Nepal; Geographical and political. The small rectangular box displays Nepal's geographic location inside Asia. Nepal is a landlocked nation in Southeast Asia that shares borders with both China and India. Lying below is the enlarged political map of Nepal. There, it makes reference of Nepal's federal provinces and districts.

Nepal, a southeast Asian country that makes up around 0.1 percent of the world's landmass, is home to 118 different ecosystems [68]. It is a biogeographical transition zone between the Paleotropic and Palaearctic biogeographical realms in the south and the Palaearctic biogeographical realms in the north [69]. Nepal is divided into three ecological regions; Mountain, hills and terai. The climate ranges from tropical to arctic in a short distance of 180 km [68]. Extreme height fluctuation (70–8848.86 m) and precipitation (up to 5500 mm yearly) [70], divergent temperatures, aspect, and humidity form a complex mosaic of ecosystems and habitat ranging from tropical forest through alpine highlands in Nepal [68]. The classification by Stainton identified 35 various forest types in Nepal [71]. Nepal is home to a vast range of flora and animals. According to Nepal's sixth national report to the Convention on Biological Diversity (2018), the country is home to over 13,000 species of flora and over 17,000 species of fauna [62]. Politically, Nepal is divided into 77 districts and 7 federal provinces (see Figure 1).

## 3 Materials and methods

### 3.1 Data collection

This checklist was created using information from previous publications in international journals, books, a computer database, and scientific records from “The World Spider Catalog” (Version-23.0). Using Google Scholar's all-in-title feature, we looked for literature discussing Nepalese spiders using keywords with Boolean operators “Spider” OR “Spiders”, “Nepal”. There were a total of 21 search results, with 14 articles and 7 citations. To find the papers listed, these citations were copied and searched in full scale in Google Chrome. Then, using the terms “Checklist of Nepalese spiders,” “List of Nepalese spiders,” “Nepalese spiders,” “Nepali spiders,” “Spiders from Nepal,” “Spiders in Nepal,” and “Spiders of Nepal”, multiple full-scale Google searches for more literature were conducted. Digital data on Nepalese spiders was collected from a government database. The World spider catalog was browsed through in order to include every article on Nepalese spiders in this study. For the last inspection, snowball referencing was used. This resulted in 94 published articles about Nepalese spiders in total. In addition, other publications, reports, and catalogs were scrutinized for accuracy.

### 3.2 Analysis

We investigated the trend in discovery of spiders from Nepal through measure of Karl Pearson’s coefficient of correlation [72]. Also, we compared our findings and examined the spider biodiversity within and outside Nepalese territory.

### 3.3 Representation

The findings were then organized as a checklist in a tabular fashion and classified according to the spider species' taxonomic family. The species' location, global distribution and bibliography are also included. The spider taxonomy is based on Version 23.0 of the World Spider Catalog (2022). The information is also displayed using descriptive statistics such as graphs and charts. The map was plotted in ArcMap version 10.4.1.

## 4 Results

**Figure 2:**
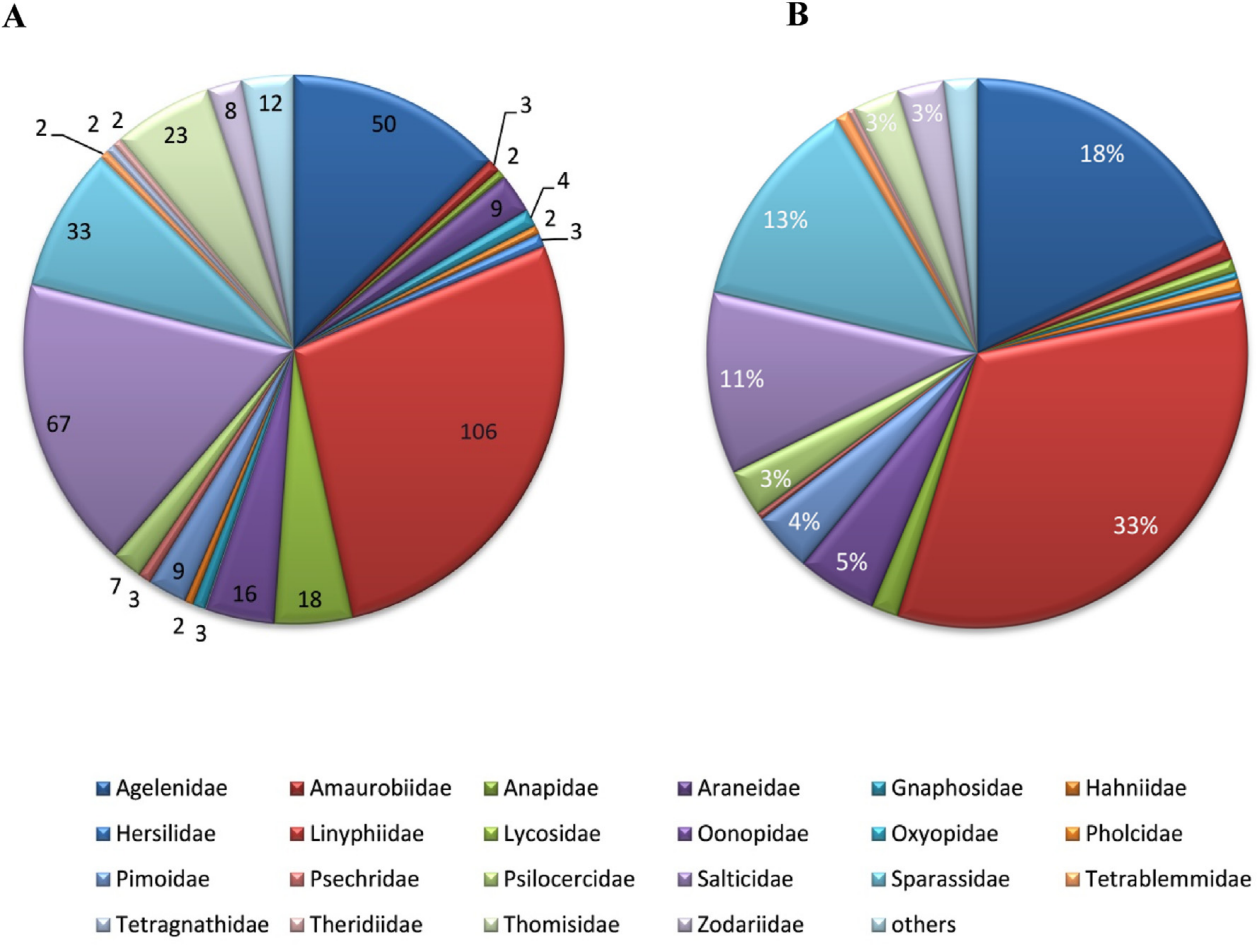
(A) Total species per family of Nepalese spiders. (B) Percentage of each family in Nepal’s endemic species.

**Figure 3:**
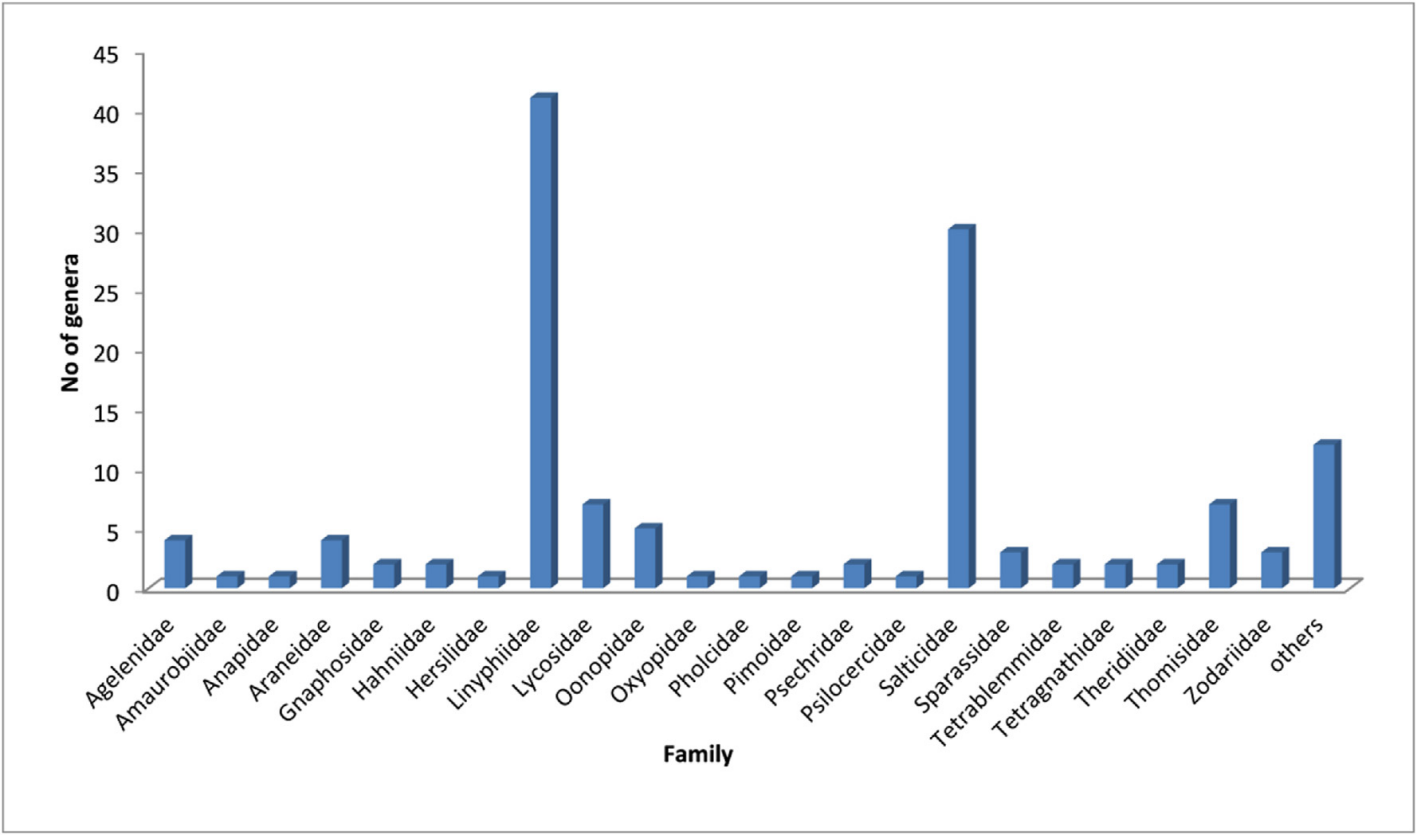
No of genera in each family of Nepalese spiders.

**Figure 4:**
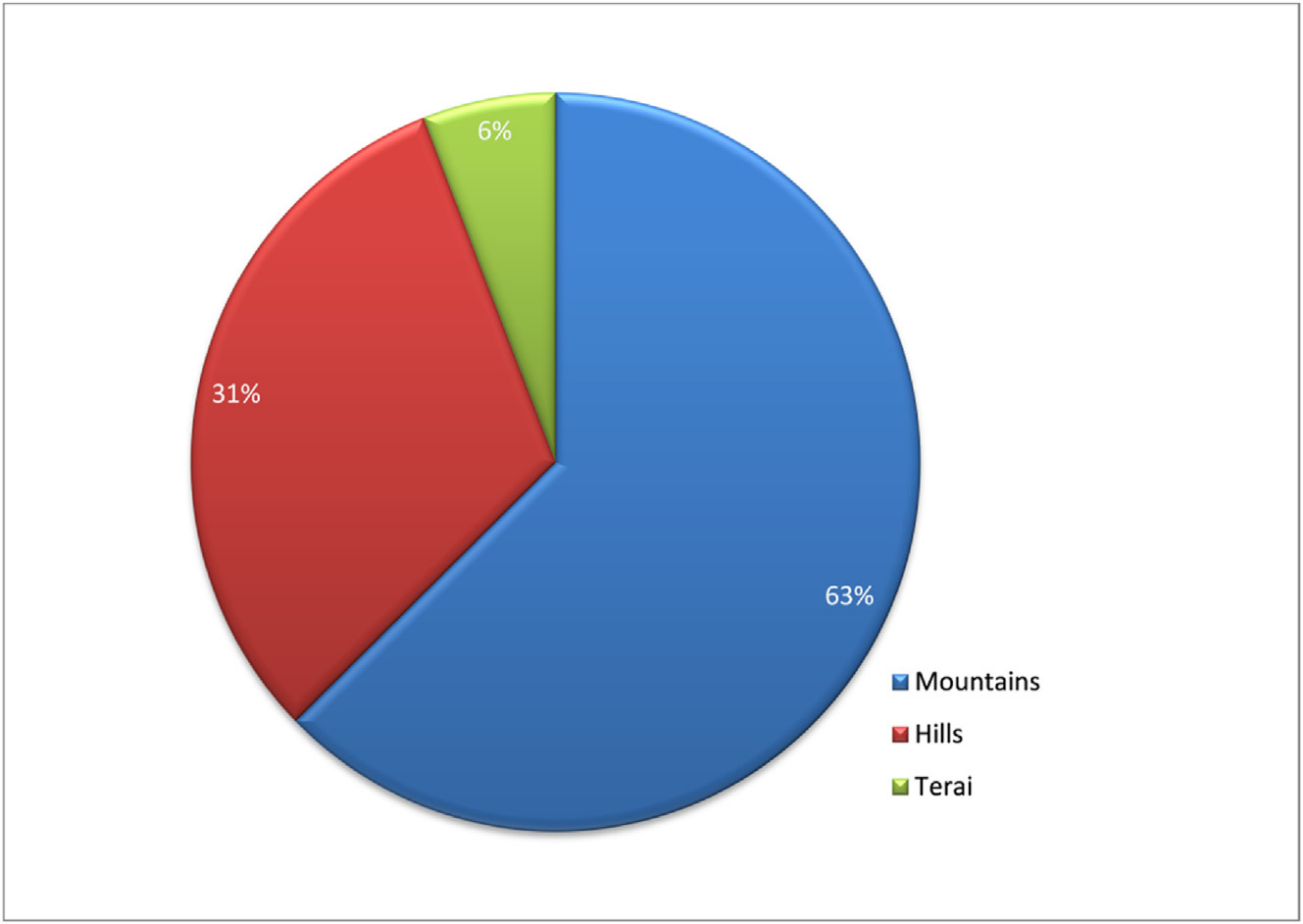
Nepalese spider species by ecological regions. **Summary of the spider fauna of Nepal.** Number of Families: 34. Number of Genera: 135. Number of Species: 386. Number of Endemic Species: 251.

**Figure 5:**
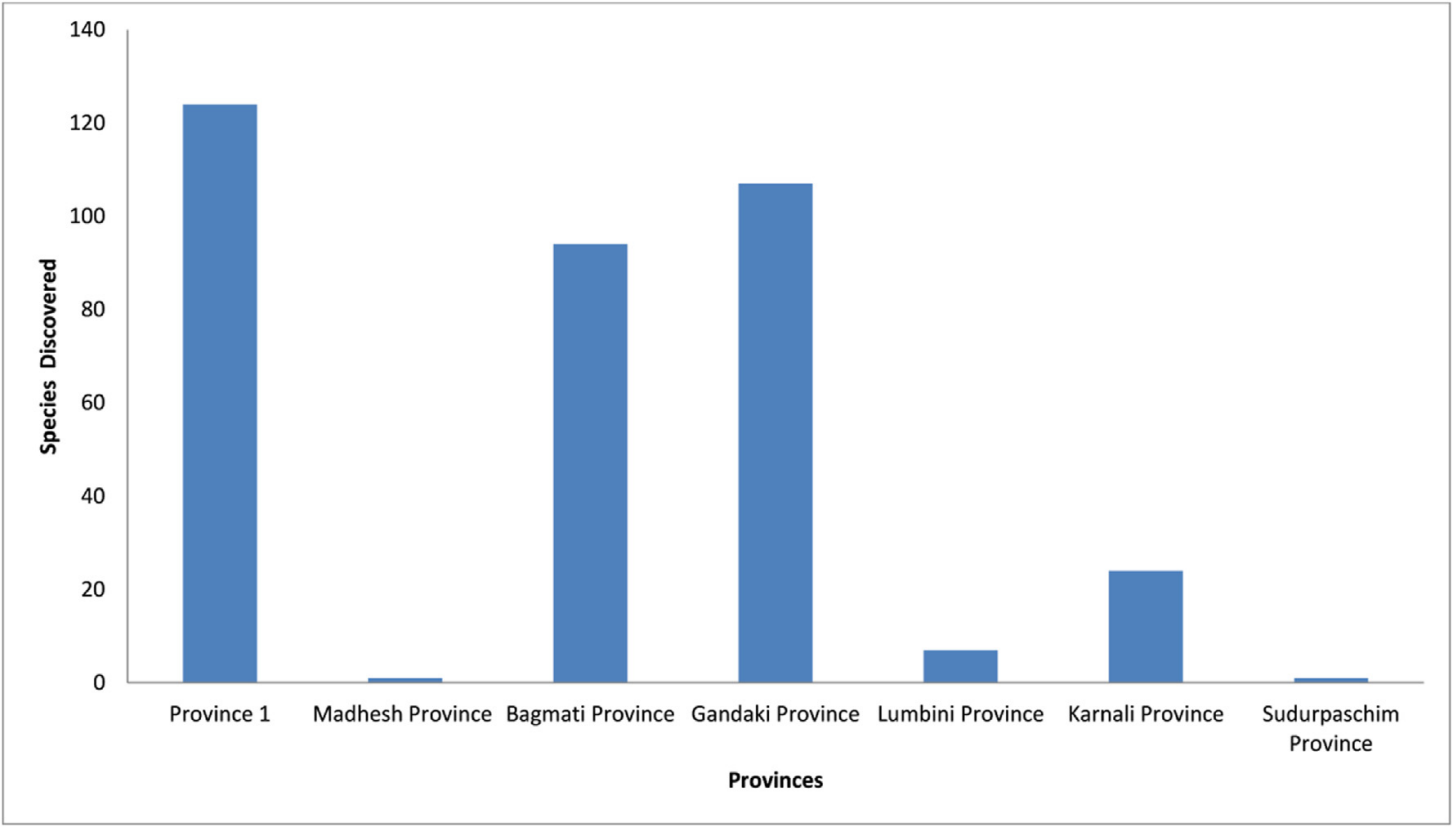
Nepalese spider species by federal provinces.

**Figure 6:**
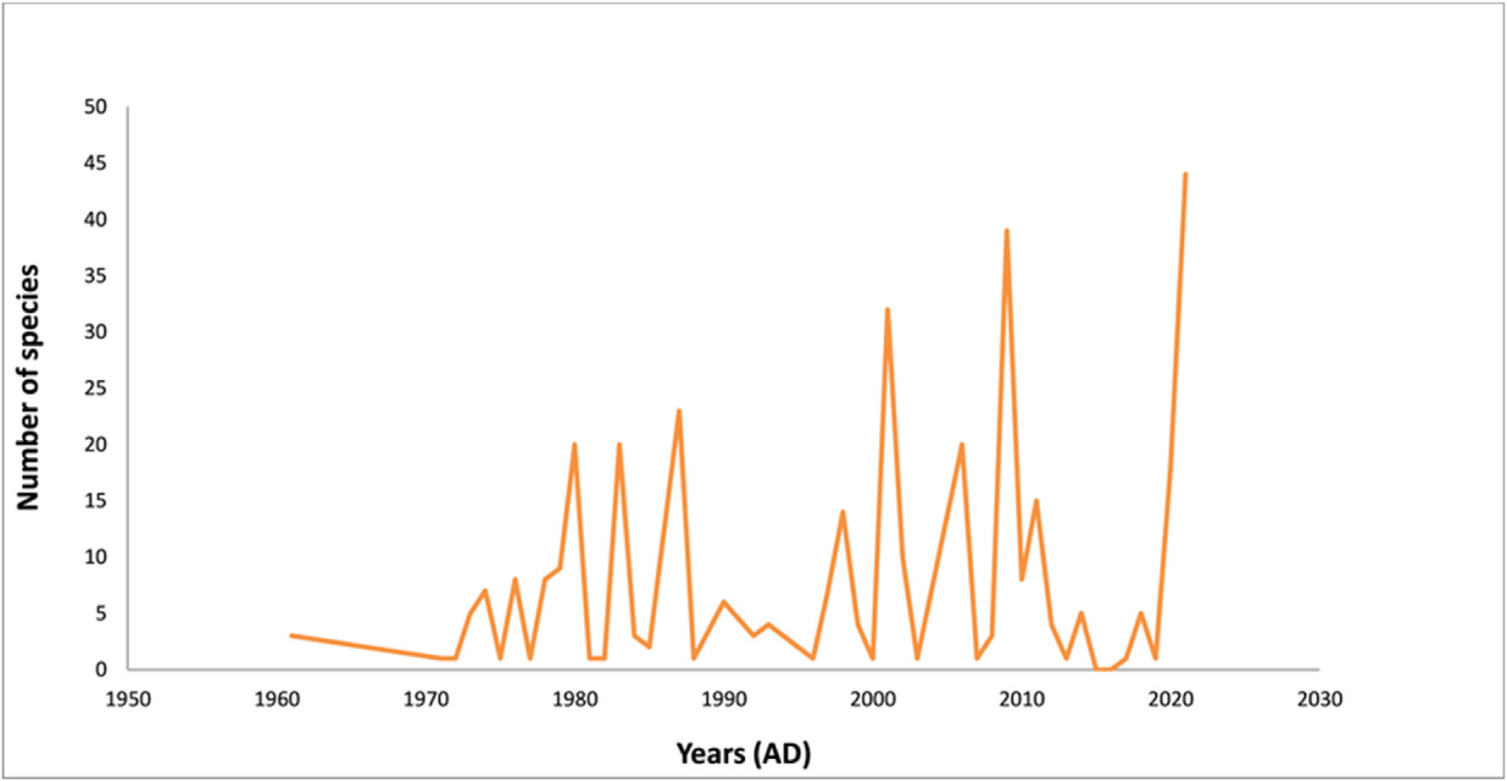
Trend of spider discoveries in Nepal.

We found 386 different spider species in Nepal, divided into 135 genera and 34 families (see Tables 1 and 2). Out of these, 251 species are endemic. This is a more than twofold increase in Nepal's spider biodiversity database. Linyphiidae and Salticidae spiders dominate accounting for 27.46 percent and 17.36 percent of total species, respectively (Figure 2, Figure 3). Nepalese spider study appears to be centered in the mountain ecological zone (63%) followed by the hills (31%) and the Terai region (6%) (Figure 4). The bulk of enlisted spiders are found in Province 1, while Sudurpaschim and Madhesh Provinces each have only one spider described (Figure 5). About 94 different articles on Nepalese spiders have been published since 1910. Karl Pearson’s correlation analysis revealed a slightly positive trend in discovery of spiders from Nepal (r =0.228). Maximum discoveries (44 species) have been been made in 2021 (Figure 6).

**Table 1:** Nepalese spider genera and species by family.

SN	Family	No.of Genera	No. of Species	No. of endemic species	Guild Structures [75]
1.	Agelenidae	4	50	46	Sheet web builders
2.	Amaurobiidae	1	3	3	Sheet web builders
3.	Anapidae	1	2	2	Orb weavers
4.	Araneidae	4	9	0	Orb weavers
5.	Ctenidae	1	1	1	Ground/other hunters
6.	Deinopidae	1	1	0	Ambush hunters
7.	Dictynidae	1	1	0	Space web builder
8.	Eresidae	1	1	0	Sheet web builders
9.	Gnaphosidae	2	4	1	Ground hunters
10.	Hahniidae	2	2	2	Sheet web builders
11.	Hersilidae	1	3	1	Sensing web builder
12.	Linyphiidae	41	106	82	Web builders/wandering
13.	Lycosidae	7	18	4	Ground hunters
14.	Mysmenidae	1	1	1	Space web builder
15.	Nesticidae	1	1	0	Space web builder
16.	Oonopidae	5	16	12	Ground hunters
17.	Oxyopidae	1	3	0	Stalkers
18.	Pholcidae	1	2	0	Space web builder
19.	Pimoidae	1	9	9	Sheet web builders
20.	Pisauridae	1	1	0	Ambush hunter
21.	Psechridae	2	3	1	Sheet web builders
22.	Psilodercidae	1	7	7	-
23.	Salticidae	30	67	27	Stalkers
24.	Scytodidae	1	1	0	Hunters
25.	Selenopidae	1	1	0	Ambush hunters
26.	Sparassidae	3	33	32	Wandering spiders
27.	Symphytognathidae	1	1	1	Orb weavers
28.	Tetrablemmidae	2	2	2	Sheet web builders
29.	Tetragnathidae	2	2	0	Orb weavers
30.	Theraphosidae	1	1	1	Sensing web builder
31.	Theridiidae	2	2	1	Space web builder
32.	Thomisidae	7	23	7	Ambush hunters
33.	Titanoecidae	1	1	1	Space web builder
34.	Zodariidae	3	8	7	Specialists

**Table 2:** Spiders of Nepal and their distribution by family

S.N	Genera	Species with Bibliography	Location (District/Province)	Distribution
**I. FAMILY AGELENIDAE** C.L. Koch, 1837				
1.	*Agelena* Walekenaer, 1837	***Agelena lukla*** [76]	Solukhumbu/ Province 1	Nepal and China
		***Agelena Sherpa*** [76]		Endemic
2.	*Draconarius* Ovtchinnikov, 1999	***Draconarius beloniformis*** [47]	Mustang/ Gandaki Province	Endemic
		***Draconarius bifarius*** [47]	Terhathum/ Province 1	Endemic
		***Draconarius brevikarenos*** [47]	Ilam/ Province 1	Endemic
		***Draconarius capitellus*** [47]	Myagdi/ Gandaki Province	Endemic
		***Draconarius communis*** [47]	Parbat/ Gandaki Province	Endemic
		***Draconarius condocephalus*** [47]	Taplejung/ Province 1	Endemic
		***Draconarius confusus*** [47]	Dolakha/ Bagmati Province	Endemic
		***Draconarius contiguus*** [47]	Dolpa/ Karnali Province	Endemic
		***Draconarius cylindratus*** [47]	Taplejung/ Province 1	Endemic
		***Draconarius dapaensis*** [47]	Mustang/ Gandaki Province	Endemic
		***Draconarius distinctus*** [47]	Panchthar / Province 1	Endemic
		***Draconarius dorsicephalus*** [47]	Dolakha/Bagmati Province	Endemic
		***Draconarius gorkhaensis*** [47]	Gorkha/ Gandaki Province	Endemic
		***Draconarius gurkha*** [47]	Solukhumbu/ Province 1	Endemic
		***Draconarius latiforus*** [47]	Sankhuwasabha / Province 1	Endemic
		***Draconarius meganiger*** [47]	Myagdi/ Gandaki Province	Endemic
		***Draconarius microcoelotes*** [47]		Endemic
		***Draconarius panchtharensis*** [47]	Panchthar/ Province 1	Endemic
		***Draconarius paraepisomos*** [47]	Mustang/ Gandaki Province	Endemic
		***Draconarius phulchokiensis*** [47]	Lalitpur/ Bagmati Province	Endemic
		***Draconarius pseudogurkha*** [47]	Solukhumbu/ Province 1	Endemic
		***Draconarius pseudomeganiger***[47]	Lalitpur/ Bagmati Province	Endemic
		***Draconarius sacculus*** [47]	Taplejung/ Province 1	Endemic
		***Draconarius schawalleri*** [47]	Gorkha/ Gandaki Province	Endemic
		***Draconarius semicirculus*** [47]	Dolakha/ Bagmati Province	Endemic
		***Draconarius seorsus*** [47]	Dolakha/ Bagmati Province	Endemic
		***Draconarius simplicifolis*** [47]	Myagdi/ Gandaki Province	Endemic
		***Draconarius spinosus*** [47]	Mustang/ Gandaki Province	Endemic
		***Draconarius subconfusus*** [47]	Panchthar/ Province 1	Endemic
		***Draconarius subepisomos*** [47]	Solukhumbu/ Province 1	Endemic
		***Draconarius subrotundus*** [47]	Sankhuwasabha / Province 1	Endemic
		***Draconarius taplejungensis*** [47]	Taplejung/ Province 1	Endemic
		***Draconarius testudinatus*** [47]	Taplejung/ Province 1	Endemic
		***Draconarius tinjuraensis*** [47]	Terhathum/ Province 1	Endemic
		***Draconarius tritos*** [47]	Taplejung/ Province 1	Endemic
		***Draconarius volutobursarius***[47]	Dolpa/ Karnali Province	Endemic
		***Draconarius wuermlii*** [47]	Taplejung/ Province 1	Nepal and Bhutan
		***Draconarius yadongensis*** [47]		Nepal and China
3.	*Himalcoelotes* Wang ,2002	***Himalcoelotes aequoreus*** [46]	Mustang/ Gandaki Province	Endemic
		***Himalcoelotes bursarius*** [46]	Sindhupalchowk/ Bagmati P.	Endemic
		***Himalcoelotes diatropos*** [46]	Rasuwa/ Bagmati P.	Endemic
		***Himalcoelotes gyirongensis***[46]	Parbat/ Gandaki P.	Nepal and China
		***Himalcoelotes martensi*** [46]	Kaski/ Gandaki Province	Endemic
		***Himalcoelotes pirum*** [46]	Manang/ Gandaki P.	Endemic
		***Himalcoelotes sherpa*** [46]	Solukhumbu/ Province 1	Endemic
		***Himalcoelotes subsherpa*** [46]	Ramechhap/ Bagmati P.	Endemic
		***Himalcoelotes syntomos*** [46]	Lalitpur/ Bagmati P.	Endemic
4.	*Tegenaria* Latreille, 1804	***Tegenaria lunakensis***[77]	Taplejung/ Province 1	Endemic
**II. FAMILY AMAUROBIIDAE** Thorell, 1870				
1.	*Himalmartensus* Wang and Zhu, 2008	***Himalmartensus ausobskyi*** [59]	Dolakha/ Bagmati P.	Endemic
		***Himalmartensus martensi*** [59]	Kathmandu/ Bagmati P.	Endemic
		***Himalmartensus nepalensis*** [59]	Rasuwa/ Bagmati P.	Endemic
**III. FAMILY ANAPIDAE** Simon, 1895				
1.	*Metanapis* Brignoli, 1981	***Metanapis montisemodi*** [7]	Myagdi/ Gandaki P.	Endemic
		***Metanapis tectimundi*** [7]	Rasuwa/ Bagmati P.	Endemic
**IV. FAMILY ARANEIDAE** Clerck, 1757				
1.	*Gasteracantha* Sundevall, 1833	***Gasteracantha kuhli*** [78]	Banke/ Lumbini P.	Nepal, India, Japan
		***Gasteracantha sanguinolenta*** [78] [[7878]]		Nepal, Africa, Yemen
2.	*Hypsosinga* Ausserer, 1871	***Hypsosinga pygmaea*** [58]	Nepal	Nepal, North America
		***Hypsosinga sanguinea*** [58]	Nepal	Nepal, Europe, North Africa
3.	*Macracantha* Simon, 1864	***Macracantha hasselti*** [78] ∗transferred from genus *Gasteracantha*	Banke/ Lumbini P.	Nepal, Pakistan, India, China
4.	*Neoscona* Simon, 1864	***Neoscona arabesca*** [58]	Nepal	Nepal, Israel, India
		***Neoscona nautical*** [58]	Nepal	Asia and pacific islands
		***Neoscona scylla*** [58]	Nepal	Nepal, Russia, China, Korea
		***Neoscona theisi*** [58]	Nepal	Nepal, Australia, India
**V. FAMILY CTENIDAE** Keyserling, 1877				
1.	*Ctenus* Walckenaer, 1805	***Ctenus martensi*** [42]	Parbat/ Gandaki Province	Endemic
**VI. FAMILY DEINOPIDAE** C.L. Koch, 1850				
1	*Asianopis* Lin and Li, 2020	***Asianopis goalparaensis*** [58] ∗Transferred from genus *Deinopis*	Nepal	Nepal and India
**VII. FAMILY DICTYNIDAE** O. Pickard-Cambridge, 1871				
*1*	*Nigma* Lehtinen, 1967	***Nigma shiprai*** [58] ∗Transferred from genus *Dictyna*	Nepal	Nepal and India
**VIII. FAMILY ERESIDAE** C.L. Koch, 1851				
1.	*Stegodyphus* Simon, 1873	***Stegodyphus sarasinorum*** [2]	Nepal	India, Sri Lanka, Nepal (Endemic to South Asia)
**IX. FAMILY GNAPHOSIDAE** Pocock, 1898				
1.	*Drassodes* Westring, 1851	***Drassodes lutescens*** [79] ∗synonym*: Phaeocedus mosambaensis*	Solukhumbu/ Province 1	Nepal, Ukraine, Caucasus
		***Drassodes phagduaensis*** [77]	Taplejung/ Province 1	Endemic
2.	*Gnaphosa* Latreille, 1804	***Gnaphosa mandschurica*** [80]	Mustang/ Gandaki P	Mongolia, China, Russia
		***Gnaphosa moerens*** [80]	Dolpa/ Karnali Province	China and Nepal
**X. FAMILY HAHNIIDAE** Bertkau, 1878				
1.	*Hahnia* C.L Koch, 1841	***Hahnia alini*** [77]	Taplejung/ Province 1	Endemic
2.	*Neoantistea* Gertsch, 1934	***Neoantistea janetscheki*** [81]	Solukhumbu/ Province 1	Endemic
**XI. FAMILY HERSILIIDAE** Thorell, 1870				
1.	*Hersilia* Audouin, 1826	***Hersilia martensi*** [82]	Gorkha/ Gandaki Province	Nepal and Thailand
		***Hersilia nepalensis*** [82]		Endemic
		***Hersilia savignyi*** [82]	Dhading/ Bagmati P.	Nepal, India, Pakistan
XII. FAMILY LINYPHIIDAE Blackwall, 1859				
1.	*Agyneta* Hull, 1911	***Agyneta bueko*** [11]	Dolpa/ Karnali Province	Endemic
		***Agynetahimalaya*** [56]	Panchthar, Province 1	Endemic
		***Agyneta jiriensis*** [11]	Dolakha/ Province 1	Endemic
		***Agyneta pakistanica*** [52]	Dailekh/ Karnali Province	Nepal and Pakistan
		***Agyneta pseudofuscipalpis*** [11]	Dolpa/ Karnali Province	Endemic
		***Agyneta yulungiensis*** [11]		Endemic
2.	*Anguliphantes* Saaristo & Tanasevitch, 1966	***Anguliphantes nepalensis*** [50] ∗Transferred from genus *Lepthyphantes*	Myagdi/ Gandaki P.	Nepal, Pakistan, India
3.	*Ascetophantes* Tanasevitch & Saaristo, 2006	***Ascetophantes asceticus*** [48] ∗Transferred from genus *Lepthyphantes*	Ilam/ Province 1	Endemic
4.	*Asthenargus* Simon and Fage, 1922	***Asthenargus thaleri*** [11]	Baglung/ Gandaki Province	Endemic
5.	*Bathyphantes* Menge, 1866	***Bathyphantes paracymbialis*** [52]	Sankhuwasabha /Province 1	Nepal, China, Laos, Myanmar, Thailand
6.	*Caviphantes* Oi, 1960	***Caviphantes pseudosaxetorum***[52]		Nepal, China , Japan
7.	*Claviphantes* Tanasevitch & Saaristo, 2006	***Claviphantes bifurcatoides*** [52] ∗Transferred from genus *Lepthyphantes*		Endemic
		***Claviphantes bifurcatus*** [48] ∗Transferred from genus *Lepthyphantes*	Parbat/ Gandaki Province	Endemic
8.	*Erigone* Audouin, 1826	***Erigone acuta*** [49]	Sankhuwasabha /Province 1	Endemic
		***Erigone atra*** [11]	Mustang/ Gandaki P.	Nepal, China, Russia
		***Erigone nepalensis*** [11]	Sindhupalchowk/ Bagmati	Endemic
		***Erigone prominens*** [52] *∗*Synonym*: Erigone ourania*	Dailekh/ Karnali Province	Nepal to China, Australia
9.	*Fistulaphantes* Tanasevitch & Saaristo, 2006	***Fistulaphantes canalis*** [52]	Sankhuwasabha /Province 1	Endemic
10.	*Gnathorium* Karsch, 1881	***Gnathorium gibberum*** [52]	Taplejung/ Province 1	Nepal, China, Korea, Japan, Russia
11.	*Gongylidiellum* Simon, 1884	***Gongylidiellum kathmanduense***[11]	Baglung/ Gandaki P.	Endemic
		***Gongylidiellum koshi*** [49]	Sankhuwasabha/Province1	Endemic
		***Gongylidiellum nepalense*** [50]	Mustang/ Gandaki P.	Nepal and India
12.	*Halorates* Hull, 1911	***Halorates crassipalpis*** [52] *∗*previously misidentified as *Collinsia japonica*	Myagdi/ Gandaki Province	Nepal and Pakistan
13.	*Helsdingenia* Saaristo and Tanasevitch, 2003	***Helsdingenia ceylonica*** [83]	Lalitpur/ Bagmati Province	Nepal, Sri Lanka (Endemic to South Asia)
14.	*Heterolinyphia* Wunderlich, 1973	***Heterolinyphia tarakotensis*** [12]	Dolpa/ Karnali Province	India and Nepal (Endemic to South Asia)
15.	*Hilaira* Simon, 1884	***Hilaira dapaensis*** [52]	Taplejung/ Province 1	Endemic
16.	*Himalafurca* Tanasevitch, 2021	***Himalafurca martensi*** [52]	Sankhuwasabha / Province 1	Endemic
		***Himalafurca schawalleri*** [52]	Taplejung/ Province 1	Endemic
17.	*Himalaphantes* Tanasevitch, 1992	***Himalaphantes grandiculus*** [52]	Panchthar/ Province 1	Endemic
		***Himalaphantes magnus*** [48]	Rasuwa/ Bagmati Province	Endemic
		***Himalaphantes martensi*** [48]	Mustang, Gandaki Province	India and Nepal
18.	*Hubertella* Platnick, 1989	***Hubertella montana*** [52]	Sindhupalchowk/ Bagmati P.	Endemic
		***Hubertella orientalis***[58]	Nepal	Endemic
		***Hubertella thankurensis*** [11]	Baglung/ Gandaki Province	Endemic
19.	*Indophantes* Saaristo and Tanasevitch, 2003	***Indophantes agamus*** [57]	Panchthar/ Province 1	Endemic
		***Indophantes digitulus*** [48]	Mustang/ Gandaki Province	Nepal, Pakistan and India
20.	*Linyphia* Latreille, 1804	***Linyphia nepalensis*** [11]	Baglung/ Gandaki Province	Endemic
21.	*Martensinus* Wunderlich, 1973	***Martensinus annulatus*** [12]	Baglung/ Gandaki Province	Endemic
		***Martensinus micronetiformis*** [11]	Mustang/ Gandaki Province	Endemic
22.	*Megalepthyphantes* Wunderlich, 1994	***Megalepthyphantes******nebulosoides*** [48] ∗transferred from genus *Lepthyphantes*	Mustang/ Gandaki Province	Central Asia, Iran
23.	*Mughiphantes* Saaristo & Tanasevitch, 1999	***Mughiphantes alticola*** [48] ∗transferred from genus *Lepthyphantes*	Mustang/ Gandaki Province	Endemic
		***Mughiphantes anachoretus*** [48] ∗transferred from genus *Lepthyphantes*		Endemic
		***Mughiphantes ancoriformis*** [52] ∗transferred from genus *Lepthyphantes*	Taplejung/ Province 1	Endemic
		***Mughiphantes bicornis*** [57]		Endemic
		***Mughiphantes cuspidatus*** [57]		Endemic
		***Mughiphantes falxus*** [57]		Endemic
		***Mughiphantes faustus*** [48] ∗transferred from genus *Lepthyphantes*	Ramechhap/ Bagmati Province	Endemic
		***Mughiphantes inermus*** [57]	Sankhuwasabha / Province 1	Endemic
		***Mughiphantes longiproper*** [57]	Taplejung/ Province 1	Endemic
		***Mughiphantes numilionis*** [48] ∗transferred from genus *Lepthyphantes*	Mustang/ Gandaki Province	Endemic
		***Mughiphantes occultus*** [48] ∗transferred from genus *Lepthyphantes*	Solukhumbu/ Province 1	Endemic
		***Mughiphantes restrictus*** [57]	Sankhuwasabha / Province 1	Endemic
		***Mughiphantes rotundatus*** [48] ∗transferred from genus *Lepthyphantes*	Myagdi/ Gandaki P.	Endemic
		***Mughiphantes setifer*** [48] ∗transferred from genus *Lepthyphantes*	Dolpa/ Karnali Province	Endemic
		***Mughiphantes setosus*** [57]	Taplejung/ Province 1	Endemic
		***Mughiphantes sherpa*** [48]	Dolpa/ Karnali Province	Endemic
		***Mughiphantes yeti*** [48] ∗ transferred from genus *Lepthyphantes*	Solukhumbu/ Province 1	Endemic
24.	*Nasoona* Locket, 1982	***Nasoona asocialis*** [52] ∗ transferred from genus *Oedothorax* ∗Previously published as *Gorbothorax ungibbus*	Kathmandu/ Bagmati Province	Nepal, China, India
		***Nasoona comata*** [53] ∗ transferred from genus *Gorbothorax*	Panchthar/ Province 1	Endemic
		***Nasoona conica*** [53] ∗ transferred from genus *Gorbothorax*	Taplejung/ Province 1	Endemic
		***Nasoona setifera*** [53] ∗ transferred from genus *Gorbothorax*	Terathum/ Province 1	Endemic
		***Nasoona wunderlichi*** [13] ∗ transferred from genus *Gorbothorax*	Dolakha/ Bagmati Province	Endemic
25.	*Nematogmus* Simon, 1886	***Nematogmus dentimanus*** [52]	Sankhuwasabha / Province 1	Nepal, Srilanka, Malaysia, Indonesia
26.	*Neriene* Blackwall, 1833	***Neriene oidedicata*** [52] ∗ transferred from genus Linyphia	Panchthar/ Province 1	Nepal, China, Russia, Korea, Japan
27.	*Nesioneta* Millidge, 1991	***Nesioneta muriensis*** [11] ∗transferred from genus *Agyneta*	Myagdi/ Gandaki Province	Endemic
28.	Oedothorax Bertkau, in Förster&Bertkau, 1883	***Oedothorax angelus*** [55]	Panchthar/ Province 1	Endemic
		***Oedothorax annulatus*** [13]	Dolakha/ Bagmati Province	Endemic
		***Oedothorax assuetus*** [55]	Kathmandu/ Bagmati P.	Endemic
		***Oedothorax clypeellum*** [55]		Endemic
		***Oedothorax coronatus*** [55]	Ilam/ Province 1	Endemic
		***Oedothorax cruciferoides*** [54]		Endemic
		***Oedothorax dismodicoides*** [52]	Myagdi/ Gandaki Province	Endemic
		***Oedothorax elongatus*** [52]	Kavre/ Bagmati Province	Endemic
		***Oedothorax falcifer*** [55]	Ilam/ Province 1	Endemic
		***Oedothorax hirsutus*** [13]	Lalitpur/ Bagmati Province	Endemic
		***Oedothorax kathmandu*** [54]	Kathmandu/ Province 1	Endemic
		***Oedothorax lineatus*** [13]	Dolakha/ Bagmati Province	Endemic
		***Oedothorax lucidus*** [13]		Endemic
		***Oedothorax malearmatus*** [55]	Panchthar/ Province 1	Endemic
		***Oedothorax mangsima*** [54]	Sankhuwasabha / Province 1	Endemic
		***Oedothorax modestus*** [55]	Panchthar/ Province 1	Endemic
		***Oedothorax savigniformis*** [55]	Taplejung/ Province 1	Endemic
		***Oedothorax sexoculatus*** [13]	Dolakha/ Bagmati Province	Endemic
		***Oedothorax sexoculorum*** [55]	Terhathum/ Province 1	Endemic
		***Oedothorax simplicithorax*** [55]	Ilam/ Province 1	Endemic
		***Oedothorax tholusus*** [55]	Kaski/ Gandaki Province	Endemic
		***Oedothorax triceps*** [54]	Sindhupalchowk/ Bagmati P.	Endemic
		***Oedothorax unicolor*** [13]	Lalitpur/ Bagmati Province	Endemic
29.	*Oia* Wunderlich, 1973	***Oia Kathmandu*** [52]	Sindhupalchowk/ Bagmati P.	Endemic
		***Oia sororia*** [52]	Myagdi/ Gandaki Province	Nepal and India
30.	*Palliduphantes* Saaristo & Tanasevitch, 2001	***Palliduphantes theosophicus*** [48] ∗ transferred from genus *Lepthyphantes*	Lalitpur/ Bagmati Province	Endemic
31.	*Paragongylidiellum* Wunderlich, 1973	***Paragongylidiellum caliginosum*** [52]	Mustang/ Gandaki P.	Nepal and India
32.	*Parbatthorax* Tanasevitch, 2019	***Parbatthorax unicornis*** [51]	Parbat/ Gandaki Province	Endemic
33.	*Piniphantes* Saaristo & Tanasevitch, 1996	***Piniphantes himalayensis*** [48]	Mustang/ Gandaki Province	Nepal and Pakistan
34.	*Porrhomma* Simon, 1884	***Porrhomma marphaense*** [11] **∗nomen dubium**	Mustang/ Gandaki Province	Endemic
35.	*Saloca* Simon, 1926	***Saloca gorapaniensis*** [11]	Mustang/ Gandaki Province	Endemic
		***Saloca khumbuensis*** [11]	Solukhumbu/ Bagmati P.	Endemic
36.	*Scotargus* Simon, 1913	***Scotargus pilosus*** [11]	Mustang/ Gandaki P.	Nepal, Europe, Algeria, Russia, Central Asia
37.	*Spiralophantes* Tanasevitch & Saaristo, 2006	***Spiralophantes mirabilis*** [57]	Sankhuwasabha / Province 1	Endemic
38.	*Tapinocyba* Simon, 1884	***Tapinocyba montivaga*** [52]	Sankhuwasabha / Province 1	Endemic
		***Tapinocyba altimontanus*** [57]		Endemic
39.	*Tenuiphantes* Saaristo & Tanasevitch, 1996	***Tenuiphantes crassus*** [57]	Taplejung / Province 1	Endemic
		***Tenuiphantes plumipes*** [48]	Gorkha/ Gandaki Province	Endemic
40.	*Tiso* Simon, 1884	***Tiso aestivus*** [52]	Taplejung / Province 1	Nepal, Canada, Japan
		***Tiso indianus*** [52]		Nepal and India
41.	*Walckenaeria* Blackwall, 1833	***Walckenaeria martensi*** [50] *∗*synonym*: Walckenaeria nepalensis*	Solukhumbu, Province 1	Nepal and India
**XIII. FAMILY LYCOSIDAE** Sundevall, 1833				
1.	*Acantholycosa* Dahl, 1908	***Acantholycosa baltoroi*** [29]	Solukhumbu/ Province 1	Nepal, India, China
2.	*Arctosa* C.L. Koch, 1847	***Arctosa janetscheki*** [27]	Kavre/ Bagmati Province	Endemic
		***Arctosa raptor*** [84]	Dolpa/ Karnali Province	Russia, Nepal, USA, Canada
3.	*Hippasa* Simon, 1885	***Hippasa greenalliae*** [58]	Nepal	Nepal, India, Sri Lanka
4.	*Hylyphantes* Simon, 1884 ∗senior synonym of genus *Erigonidium*	***Hylyphantes graminicola*** [58]	Nepal	Nepal, Europe, Russia, China
5.	*Lycosa* Gravely, 1924	***Lycosa kempi*** [27]	Dolakha/ Bagmati Province	Nepal, Pakistan, India, China
6.	*Pardosa* C.L. Koch, 1847	***Pardosa bifasciata*** [27] *∗*previously published as *Pardosa thaleri*	Solukhumbu/ Province 1	Nepal, Europe, Turkey, Russia, China
		***Pardosa birmanica*** [27]	Solukhumbu/ Province 1	Nepal, Myanmar
		***Pardosa fletcheri*** [29]	Myagdi/ Gandaki P.	Nepal, Pakistan, India
		***Pardosa martensi*** [29]	Dolpa/ Karnali Province	Endemic
		***Pardosa mongolica*** [28]		Nepal, Russia, Mongolia,China
		***Pardosa orealis*** [28]		Endemic
		***Pardosa pseudoannulata*** [58] *∗Synonym: Lycosa pseudoannulata*	Nepal	Nepal, Pakistan, China, India, Bhutan, Japan, Indonesia
		***Pardosa pusiola*** [2]	Nepal	Nepal, Bhutan, India
		***Pardosa sumatrana*** [27]	Solukhumbu/ Province 1	Nepal, Bhutan, India
		***Pardosa sutherlandi*** [29]	Parbat/ Gandaki P.	Nepal, Bhutan, India
		***Pardosa tridentis*** [27]	Solukhumbu/ Province 1	Nepal, India, Kashmir
7.	*Trochosa* C.L. Koch, 1847	***Trochosa gravelyi*** [27]	Kavre/ Bagmati Province	Endemic
**XIV. FAMILY MYSMENIDAE** Petrunkevitch, 1928				
1.	*Iardinis* Simon, 1899	***Iardinis martensi*** [7]	Dolakha/ Bagmati P.	Endemic
**XV. FAMILY NESTICIDAE** Simon, 1894				
1.	*Nesticella* Lehtinen &Saaristo, 1980	***Nesticella nepalensis*** [85]	Dolakha/ Bagmati P.	Nepal, China, India
**XVI. FAMILY OONOPIDAE** Simon, 1890				
1.	*Brignolia* Dumitrescu and Georgescu, 1983	***Brignolia ankhu*** [60]	Dhading/ Bagmati P.	Endemic
		***Brignolia assam*** [60]	Nuwakot/ Bagmati P.	Nepal and India
		***Brignolia sukna*** [60]	Ilam/ Province 1	Nepal and India
2.	*Camptoscaphiella* Caporiacco, 1934	***Camptoscaphiella gunsa*** [86]	Taplejung/ Province 1	Nepal and India
		***Camptoscaphiella martensi*** [86]	Mustang/ Gandaki P.	Endemic
		***Camptoscaphiella nepalensis*** [86]	Parbat/ Gandaki P.	Endemic
		***Camptoscaphiella panchthar*** [86]	Panchthar/ Province 1	Endemic
		***Camptoscaphiella silens*** [86]	Solukhumbu/ Province 1	Endemic
		***Camptoscaphiella strepens*** [86]	Gorkha/ Gandaki P.	Endemic
		***Camptoscaphiella taplejung*** [86]	Taplejung/ Province 1	Endemic
3.	*Himalayana* Grismado, 2014	***Himalayana castanopsis*** [61]	Ilam/ Province 1	Endemic
		***Himalayana kathmandu*** [61]	Kathmandu/ Bagmati P.	Endemic
		***Himalayana martensi*** [61]	Manang/ Gandaki P.	Endemic
		***Himalayana parbat*** [61]	Parbat/ Gandaki P.	Endemic
4.	*Prethopalpus* Baehr et al., 2012	***Prethopalpus ilam*** [87]	Ilam/ Province 1	Endemic
5.	*Trilacuna* Tong & Li, 2007	***Trilacuna bangla*** [61]	Sindhupalchowk/ Bagmati Province	Nepal and India
**XVII. FAMILY OXYOPIDAE** Thorell, 1869				
1	*Oxyopes* Latreille, 1804	***Oxyopes javanus*** [58]	Nepal	Nepal, China, India
		***Oxyopes lineatus*** [58]	Nepal	Nepal, Europe, Turkey ,Russia
		***Oxyopes sertatus*** [58]	Nepal	Nepal, China, Korea, Japan
**XVIII. FAMILY PHOLCIDAE** C.L. Koch, 1850				
1.	*Pholcus* Walckenaer, 1805	***Pholcus calligaster*** [88]	Parsa/ Madhesh Province	Nepal and Myanmar
		***Pholcus zham*** [88]	Sankhuwasabha/Province1	Nepal and China
**XIX. FAMILY PIMOIDAE** Wunderlich, 1986				
1.	*Pimoa* Chamberlin & Ivie, 1943	***Pimoa daman*** [67]	Makwanpur/ Bagmati P.	Endemic
		***Pimoa khaptad*** [67]	Bajhang/ Sudurpashim P.	Endemic
		***Pimoa koshi*** [67]	Sankhuwasabha/Province1	Endemic
		***Pimoa mechi*** [67]	Taplejung/ Province 1	Endemic
		***Pimoa mude*** [67]	Sindhupalchowk/Bagmati	Endemic
		***Pimoa nematoides*** [88]	Dolakha/ Bagmati P.	Endemic
		***Pimoa phaplu*** [67]	Solukhumbu/ Province 1	Endemic
		***Pimoa rara*** [67]	Mugu/ Karnali Province	Endemic
		***Pimoa sinuosa*** [88]	Kaski/ Gandaki Province	Endemic
**XX. FAMILY PISAURIDAE** Simon, 1890				
1.	*Perenethis* L. Koch, 1878	***Perenethis sindica*** [89]	Taplejung/ Province 1	India, Sri Lanka, Nepal, China
**XXI. FAMILY PSECHRIDAE** Simon, 1890				
1.	*Psechrus* Thorell, 1878	***Psechrus himalayanus*** [90]	Rolpa/ Lumbini Province	India, Nepal
		***Psechrus marsyandi*** [45]	Lamjung/ Gandaki P.	Endemic
2.	*Fecenia* Simon, 1887	***Fecenia protensa*** [58] ∗Synonym: *Facenia nicobarensis*	Nepal	Nepal, Thailand, Vietnam, Brunei, Malaysia, India
∗*Fecinia nicobarensis* was transferred from genus *Psechrus* (Thorell, 1878)				
**XXII. FAMILY PSILOCERCIDAE** Machado, 1951				
1.	*Leclercera* Deeleman-Reinhold, 1995	***Leclercera ekteenensis*** [64]	Panchthar/ Province 1	Endemic
		***Leclercera machadoi*** [9]	Baglung/ Gandaki P.	Endemic
		***Leclercera mulcata*** [9] *∗*transferred from genus *Psiloderces*	Kathmandu/ Bagmati P.	Endemic
		***Leclercera nagarjunensis*** [63]		Endemic
		***Leclercera niuqu*** [63]	Panchthar/ Province 1	Endemic
		***Leclercera sidai*** [63]	Ilam/ Province 1	Endemic
		***Leclercera zhaoi*** [63]		Endemic
*∗*Psilocercidae, a sub family of Ochyroceratidae was raised to family by Wunderlich (2008)				
**XXIII. FAMILY SALTICIDAE** Blackwall, 1841				
1.	*Asemonea* O Pickard-Cambridge, 1869	***Asamonea tenuipes*** [66]	Chitwan/ Bagmati Province.	Nepal, Sri Lanka, India, Myanmar, Vietnam, Singapore
2.	*Bianor* *Peckham and Peckham, 1886*	***Bianor albobimaculatus*** [91]	Manang/ Gandaki P.	Nepal, Iran, Pakistan India
		***Bianor tortus*** [37]	Ilam/ Province 1	Nepal and India
3.	*Brettus* Thorell, 1895	***Brettus anchorum***[36]	Gorkha/ Gandaki Province	Nepal and India
4.	*Carrhotus* Thorell, 1891	***Carrhotus assam*** [91]	Kaski/ Gandaki Province	Nepal and India
		***Carrhotus catagraphus*** [34]	Gorkha/ Gandaki Province	Endemic
		***Carrhotus erus*** [91]	Kaski/ Gandaki P.	Nepal and India
		***Carrhotus operosus*** [34]	Mustang/ Gandaki P.	Endemic
		***Carrhotus s-bulbosus*** [32]	Sankhuwasabha/Province1	Endemic
		***Carrhotus sannio*** [91]	Myagdi/ Gandaki	Nepal, China, India
		***Carrhotus viduus*** [34]		Nepal, China. India, Iran
5.	*Chalcoscirtus* Bertkau, 1880	***Chalcoscirtus jiricus*** [22] *∗*transferred from genus *Euophrys*	Dolakha/ Bagmati Province	Endemic
		***Chalcoscirtus martensi*** [22]	Mustang/ Gandaki P.	Nepal, India and China
6.	*Chinattus* Logunov, 1999	***Chinattus chichila*** [92]	Sankhuwasabha/Province1	Endemic
		***Chinattus validus*** [93]	Myagdi/ Gandaki P.	Nepal,Bhutan,China
7.	*Chrysilla* Thorell, 1887	***Chrysilla volupe*** [66]	Chitwan/ Bagmati Province	Nepal, Bhutan, India, Sri Lanka
8.	*Epeus* Peckham and Peckham*, 1886*	***Epeus exdomus*** [94]	Kathmandu/ Bagmati P.	Endemic
		***Epeus indicus*** [35]	Nuwakot/ Bagmati P.	Nepal and India
9.	*Epocilla* Thorell, 1887	***Epocilla aurantiaca*** [66]	Chitwan/ Bagmati Province.	Nepal, Sri Lanka, Malaysia, Vietnam, India
10.	*Euophrys* C. L. Koch, 1834	***Euophrys dhaulagirica*** [22]	Mustang/ Gandaki P.	Endemic
		***Euophrys nepalica*** [22]	Myagdi/ Gandaki P.	Nepal and China
		***Euophrys omnisuperstes*** [5]	Sankhuwasabha/Province1	Nepal and India
		***Euophrys yulungensis*** [22]	Dolpa/ Karnali Province	China and Nepal
11.	*Habrocestoides* Prószyn'ski, 1992	***Habrocestoides phulchokiensis*** [95]	Lalitpur/ Bagmati Province	Endemic
12.	*Harmochirus* Simon, 1885	***Harmochirus zabkai*** [96]	Kathmandu/ Bagmati P.	India, Nepal, Vietnam
13.	*Hyllus* C.L. Koch, 1846	***Hyllus semicupreus*** [66]	Chitwan/ Bagmati Province.	Nepal, Sri Lanka, India
14.	*Icius* Simon, 1876	***Icius alboterminus*** [66]		Nepal and India
15.	*Nepalicus* Blackwall, 1841	***Nepalicius nepalicus*** [97] ∗Transferred from genus *Pseudicius*	Kathmandu/ Bagmati Province	Nepal and India
16.	*Orientattus* Caleb, 2020	***Orientattus minutes*** [23] ∗*O.minutes* was transferred from genus *Pancorius*	Gorkha/ Gandaki Province	Nepal
17.	*Pancorius* Simon, 1902	***Pancorius armatus*** [39]	Parbat/ Gandaki Province	Endemic
		***Pancorius cadus*** [39]	Taplejung/ Province 1	Endemic
		***Pancorius kaskiae*** [23]	Kaski/ Gandaki Province	Endemic
		***Pancorius magnus*** [39]	Ilam/ Province 1	Nepal, China, India
		***Pancorius urnus*** [39]	Ilam/ Province 1	Endemic
18.	*Phaeacius* Simon, 1900	***Phaeacius fimbriatus*** [36]	Sankhuwasabha/Province1	Nepal, Indonesia, Java
		***Phaeacius saxicola*** [98]	Taplejung/ Province 1	Endemic
		***Phaeacius wanlessi*** [36]	Sankhuwasabha/Province1	Nepal, Sri Lanka
19.	*Phintella* Strand, 1906	***Phintella suavis*** [2]	Nepal	Nepal to Malaysia
		***Phintella vittata*** [66]	Chitwan/ Bagmati P.	Nepal, China, India
20.	*Plexippoides* Prószyn'ski, 1984	***Plexippoides tristis*** [99]	Mustang/ Gandaki P.	Endemic
21.	*Plexippus* C.L. Koch, 1846	***Plexippus paykulli*** [23]	Myagdi/ Gandaki P.	Asia, Africa, America, Europe
		***Plexippus petersi*** [23]	Kaski/ Gandaki Province	Asia, Africa and Pacific islands
		***Plexippus pokharae*** [23]		Endemic
22.	*Portia* Karsch, 1878	***Portia fimbriata*** [36]	Kathmandu/ Bagmati P.	Nepal, Sri Lanka, Taiwan to Australia
23.	*Ptocasius* Simon, 1885	***Ptocasius nepalicus*** [20] *Synonym: Yaginumaella nepalica*	Mustang/ Gandaki Province	Nepal and China
		***Ptocasius tenzingi*** [20] *Synonym: Yaginumaella tenzingi*	Solukhumbu/ Province 1	Endemic
		***Ptocasius thakkholaicus*** [20] *Synonym: Yaginumaella thakkholaica*	Mustang/ Gandaki Province	Nepal and China
24.	*Rhene* Thorell, 1869	***Rhene flavicomans*** [33]	Sankhuwasabha/Province1	Nepal, Bhutan, India, Thailand
		***Rhene phuntsholingensis*** [33]		Nepal, Bhutan
25.	*Siler* Simon, 1889	***Siler cupreus*** [66]	Chitwan/ Bagmati P.	Nepal, China, Taiwan, Korea, Japan
26.	*Sitticus* Simon 1901	***Sitticus niveosignatus*** [21]	Dolpa/ Karnali Province	Nepal to China
27.	*Stenaelurillus* Simon,1886	***Stenaelurillus triguttatus*** [100]	Narayangadh/ Bagmati P.	Nepal and China
28.	*Synagelides* Strand, 1906	***Synagelides bagmaticus*** [101]	Bhaktapur/ Bagmati P.	Endemic
		***Synagelides gosainkundicus*** [101]	Rasuwa/ Bagmati P.	Endemic
		***Synagelides kosi*** [101]	Ramechhap/ Bagmati P.	Endemic
		***Synagelides martensi*** [101] Synonyms*: Synagelides dhaulagiricus* ,*Synagelides himalaicus, Synagelides jiricus, Synagelides thodungus & Synagelides wyszynskii*	Dolpa/ Karnali Province	Endemic
		***Synagelides nepalensis*** [24]		Endemic
		***Synagelides nishikawai*** [25]	Myagdi/ Gandaki P.	Endemic
		***Synagelides oleksiaki*** [24] *Synonym: Synagelides gorapanicus*	Ramechhap/ Bagmati P.	Endemic
		***Synagelides tukchensis*** [24]	Mustang/ Gandaki P.	Endemic
		***Synagelides ullerensis*** [24]	Parbat/ Gandaki P.	Endemic
		***Synagelides walesai*** [24]	Lalitpur/ Bagmati P.	Endemic
29.	*Telamonia* Thorell,1887	***Telamonia dimidiata*** [66]	Chitwan/ Bagmati P.	Nepal, Bhutan, Malaysia
		***Telamonia festiva*** [66]	Chitwan/ Bagmati P.	Nepal, China, India
30.	*Thyene* Simon,1885	***Thyene bivittata*** [38]	Kathmandu/ Bagmati P.	Nepal, China, Pakistan
		***Thyene typica*** [38]	Sankhuwasabha /Province 1	Endemic
		***Thyene yuxiensis*** [38]	Tanahu/ Gandaki P.	Nepal and China
**XXIV. FAMILY SCYTODIDAE** Blackwall, 1864				
1.	*Scytodes* Latreille, 1804	***Scytodes mawphlongensis*** [10]	Lalitpur/ Bagmati Province	Nepal and India
**XXV. FAMILY SELENOPIDAE** Simon, 1897				
1.	*Makdiops* Crews and Harvey,2011	***Makdiops montigena*** [102]	Chitwan/ Bagmati Province	Nepal and India
**XXVI. FAMILY SPARASSIDAE** Bertkau, 1872				
1.	*Bhutaniella* Jäger, 2000	***Bhutaniella hillyardi*** [41]	Sankhuwasabha/Province1	Endemic
		***Bhutaniella rollardae*** [43]	Pyuthan/ Lumbini P.	Endemic
2.	*Olios* Walckenaer,1837	***Olios rossetti*** [44]	Kavre/ Bagmati Province	Nepal, India, Pakistan
3.	*Pseudopoda* Jäger, 2000	***Pseudopoda albolineata*** [82]	Myagdi/ Gandaki P.	Endemic
		***Pseudopoda alta*** [43]	Kaski/ Gandaki Province	Endemic
		***Pseudopoda ausobskyi*** [43]	Ilam/ Province 1	Endemic
		***Pseudopoda brauni*** [43]	Taplejung/ Province 1	Endemic
		***Pseudopoda chauki*** [43]	Terathum/ Province 1	Endemic
		***Pseudopoda chulingensis*** [43]	Gorkha/ Gandaki Province	Endemic
		***Pseudopoda cuneata*** [43]	Myagdi/ Gandaki P.	Endemic
		***Pseudopoda dama*** [43]	Jhapa/ Province 1	Endemic
		***Pseudopoda damana*** [43]	Makwanpur/ Bagmati P.	Endemic
		***Pseudopoda dhulensis*** [43]	Baglung/ Gandaki P.	Endemic
		***Pseudopoda diversipunctata*** [43]	Kaski/ Gandaki Province	Endemic
		***Pseudopoda everesta*** [43]	Solukhumbu/ Province 1	Endemic
		***Pseudopoda grasshoffi*** [43]	Sankhuwasabha /Province 1	Endemic
		***Pseudopoda heteropodoides*** [43]	Taplejung/ Province 1	Endemic
		***Pseudopoda huberti*** [43]	Pyuthan/ Lumbini P.	Endemic
		***Pseudopoda hyatti*** [43]	Myagdi/ Gandaki P.	Endemic
		***Pseudopoda jirensis*** [43]	Dolakha/ Bagmati P.	Endemic
		***Pseudopoda kalinchoka*** [43]	Dolakha/ Bagmati P.	Endemic
		***Pseudopoda khimtensis*** [43]	Ramechhap/ Bagmati P.	Endemic
		***Pseudopoda latembola*** [43]	Manang/ Gandaki P.	Endemic
		***Pseudopoda marmoreal*** [43]	Kaski/ Gandaki Province	Endemic
		***Pseudopoda martensi*** [43]	Mustang/ Gandaki P.	Endemic
		***Pseudopoda martinae*** [43]	Rasuwa/ Bagmati P.	Endemic
		***Pseudopoda monticola*** [43]	Lalitpur/ Bagmati P.	Endemic
		***Pseudopoda schawalleri*** [43]	Panchthar/ Province 1	Endemic
		***Pseudopoda sinopodoides*** [43]	Kathmandu/ Bagmati P.	Endemic
		***Pseudopoda tinjura*** [43]	Tehrathum/ Province 1	Endemic
		***Pseudopoda triapicata*** [43]	Ilam/ Province 1	Endemic
		***Pseudopoda trisuliensis*** [43]	Rasuwa/ Bagmati P.	Endemic
		***Pseudopoda varia*** [43]	Taplejung/ Province 1	Endemic
**XXVII. FAMILY SYMPHYTOGNATHIDAE** Hickman, 1931				
1	*Iardinis* Simon,1899	***Iardinis martensi*** [7]	Dolakha/ Bagmati Province	Endemic
**XXVIII. FAMILY TETRABLEMMIDAE** O.P-Cambridge, 1873				
1.	*Brignoliella* Shear, 1978	***Brignoliella martensi*** [8]	Lalitpur/ Bagmati Province	Endemic
2.	*Tetrablemma* O.P.-Cambridge, 1873	***Tetrablemma phulchoki*** [14]		Endemic
**XXIX. FAMILY TETRAGNATHIDAE** Menge, 1866				
1.	*Leucauge* White,1841	***Leucauge decorata*** [58]	Nepal	Nepal, Japan, Thailand, Bangladesh, China, India
2.	*Tetragnatha* Latreille,1804	***Tetragnatha bogotensis*** [103] Synonym: *Tetragnatha boydi*	Nepal	Nepal, Spain, Mexico to Paraguay
**XXX. FAMILY THERAPHOSIDAE** Thorell, 1870				
1.	*Haplocosmia* Schmidt & von Wirth, 1996	***Haplocosmia nepalensis*** [104]	Kaski/ Gandaki Province	Endemic
**XXXI. FAMILY THERIDIIDAE** Sundevall, 1833				
1.	*Carniella* Thaler & Steinberger	***Carniella nepalensis*** [105]	Taplejung/ Province 1	Endemic
2.	*Lactrodectus* Walckenaer,1805	***Lactrodectus elegans*** [65]	Gorkha/ Gandaki Province	Nepal, China, Japan , India, Myanmar
**XXXII. FAMILY THOMISIDAE** Sundevall, 1833				
1.	*Bassaniodes* Pocock, 1903	***Bassaniodes dolpoensis*** [15] ∗transferred from genus *Xysticus*	Dolpa/ Karnali province	Nepal and China
2.	*Lysiteles* Simon, 1895	***Lysiteles annapurnus*** [18]	Kaski/ Gandaki Province	Endemic
		***Lysiteles himalayensis*** [18]	Myagdi/ Gandaki Province	Bhutan, Nepal
		***Lysiteles lepusculus*** [18]	Mustang/ Gandaki P.	Endemic
		***Lysiteles maius*** [18]	Baitadi/ Gandaki P.	Russia, Nepal to Japan
		***Lysiteles montivagus*** [18]	Mustang/ Gandaki P.	Endemic
		***Lysiteles niger*** [18]	Makwanpur/ Bagmati P.	Bhutan, Nepal
		***Lysiteles parvulus*** [18]	Myagdi/ Gandaki Province	Endemic
		***Lysiteles saltus*** [18]		Bhutan , Nepal, China
3.	*Monaeses* Thorell, 1869	***Monaeses aciculus*** [16]	Taplejung/ Province 1	Nepal to Japan, Philippines
4.	*Psammitis* Menge, 1876	***Psammitis nepalhimalaicus*** [15] ∗transferred from genus *Xysticus*	Dolakha/ Bagmati Province	Endemic
		***Psammitis potamon*** [15] ∗transferred from genus *Xysticus*	Myagdi/ Gandaki Province	Endemic
		***Psammitis simplicipalpatus*** [15] ∗transferred from genus *Xysticus*	Dolpa/ Karnali Province	Nepal and Bhutan
5.	*Runcinia* Simon, 1875	***Runcinia roonwali*** [58]	Nepal	Nepal and India
		***Runcinia insecta*** [58] *∗*previously published as *Thomisus cherapunjeus*	Nepal	Asia, Africa, Australia
6.	*Stiphropus* Gerstäcker, 1873	***Stiphropus soureni*** [17]	Kavre/ Bagmati P.	India, Nepal, Bhutan
7.	*Xysticus* C.L. Koch, 1835	***Xysticus alpinistus*** [15]	Dolakha/ Bagmati P.	Nepal, China
		***Xysticus cristatus*** [15]	Mustang/ Gandaki P.	Nepal, Kazakhstan, Iran
		***Xysticus croceus*** [2]	Nepal	India, Nepal, Bhutan, China
		***Xysticus elephantus*** [15]	Dolpa/ Karnali Province	Nepal, China
		***Xysticus martensi*** [15]		Endemic
		***Xysticus roonwali*** [106]	Solukhumbu/ Province 1	Nepal, India
		***Xysticus cf sikkimus*** [15]	Mustang/ Gandaki P.	Nepal, China, India
**XXXIII. FAMILY TITANOECIDAE** Lehtinen, 1967				
1.	*Anuvinda* Lehtinen, 1967	***Anuvinda milloti*** [107] ***∗***transferred from genus *Amaurobius*	Chitwan/ Bagmati P.	Endemic
**XXXIV. FAMILY ZODARIIDAE** Thorell, 1881				
1.	*Mallinella* Strand, 1906	***Mallinella erratica*** [19] ∗transferred from genus *Storena*	Ilam/ Province 1	Endemic
		***Mallinella martensi*** [19] ∗transferred from genus *Storena*	Mustang/ Gandaki Province	Endemic
		***Mallinella nepalensis*** [19] ∗transferred from genus *Storena*	Rasuwa/ Bagmati Province	Endemic
		***Mallinella uncinata*** [19] ∗transferred from genus *Storena*	Kaski/ Gandaki Province	Endemic
2.	*Suffasia* Jocqué, 1991	***Suffasia kanchenjunga*** [31]	Ilam/ Province 1	Endemic
		***Suffasia martensi*** [31]	Ilam/ Province 1	Endemic
		***Suffasia tumegaster*** [108]	Lalitpur/ Bagmati P.	Endemic
3.	*Tropizodium* Jocque & Churchill, 2005	***Tropizodium bengalensis*** [58] *∗*transferred from genus *Lutica*	Nepal	Nepal and India

This checklist contains taxonomic upgrades as well as corrections to past misidentifications. To avoid recurrence, synonymous species are sorted. Seven Tetrablemmidae species (*Tetrablemma elongata*, *Tetrablemma laboriosa*, *Tetrablemma mandibulata*, *Tetrablemma maxillosa*, *Tetrablemma phulchoki*, *Tetrablemma straminea*, *Tetrablemma virescens*, *Theridiosoma* sp) and two Synagelides species (*Synagelides wangdicus* and *Synagelides wuermlii*) enlisted earlier [30, 58] are omitted in this checklist due to lack of published references or collected specimens. Similarly, some enlistments from earlier lists are excluded due to unidentified specific trait. The checklist has also taken into account previous misidentifications, synonyms, and taxonomic transfers of several spider species.

## 5 Discussion

With 386 species of spiders, Nepal has about five times more species-to-area ratio (0.00262) than its neighbouring countries; China (0.000546) and India (0.000512) having 5249 and 1686 spider species each [73, 74]. It accounts for 16.79% of spiders of South Asia (2299 species) and 0.77% of total spiders in the world [2, 30]. The family Linyphiidae dominates the spider inventory of Nepal, although Salticids lead the Chinese and Indian catalogues. With 63% of total spiders enlisted, maximum expeditions have been focused on Mountain ecological region of Nepal. In Nepalese spiders, there are conspicuous Himalayan radiations. Deeply separated valleys and a plethora of mountain ranges preventing ground-dwelling arthropods from spreading quickly from one valley chain to the next, has resulted in the evolution of several species [47]. The diversity of coelotine spiders in Nepal astounded the authors; Wang and Martens [47]. Local species of genera *Draconarius*, *Pseudopoda*, and *Bhutaniella* have particularly striking traits [43, 47]. The existence of *Euophrys omnisuperstes* amid snow and stony debris at a height of 6700 m above sea level is intriguing.

The study of Nepal's endemic Himalayan spider species has got a good attention, but the lush lower vegetation has been overlooked. Vast swaths of biologically significant land have remained mostly unexplored. Out of 77 districts of Nepal, 39 have not been explored a bit for spider diversity. Since 1910, there have been only 94 publications on Nepalese spiders. There is a weak positive Karl Pearson’s coefficient of correlation (r = 0.228) between years and new spiders discovered in Nepal. A simple keyword search {allintitle: spider “Country name”} yields about 40 times less results on google scholar for Nepal than those for China and India. These clearly indicate a significant research gap. Scholars from around the world appear to be curious but Nepalese have played a modest role in spider studies. Also, a 100% research focus has been on baseline surveys. With growing global interests on spider webs, ecology and venom, other thematic areas should be covered as well. Nepal thus seems a promising land for spider diversity. Further explorations might significantly boost global spider inventory. The authors thus invite and encourage researchers from all around the world to investigate Nepalese spiders.

## Declarations

### Author contribution statement

All authors listed have significantly contributed to the development and the writing of this article.

### Funding statement

This research did not receive any specific grant from funding agencies in the public, commercial, or not-for-profit sectors.

### Data availability statement

Data included in article/supplementary material/referenced in article.

### Declaration of interests statement

The authors declare no conflict of interest.

### Additional information

No additional information is available for this paper.
